# miR-224-5p Carried by Human Umbilical Cord Mesenchymal Stem Cells-Derived Exosomes Regulates Autophagy in Breast Cancer Cells via HOXA5

**DOI:** 10.3389/fcell.2021.679185

**Published:** 2021-05-21

**Authors:** Yichao Wang, Pan Wang, Lei Zhao, Xiaoying Chen, Zhu Lin, Ling Zhang, Zhaoyun Li

**Affiliations:** ^1^Department of Clinical Laboratory Medicine, Taizhou Central Hospital (Taizhou University Hospital), Taizhou City, China; ^2^Department of Ultrasound, Taizhou Central Hospital (Taizhou University Hospital), Taizhou City, China; ^3^Department of Obstetrics and Gynecology, Taizhou Central Hospital (Taizhou University Hospital), Taizhou City, China

**Keywords:** breast cancer, human umbilical cord mesenchymal stem cells, exosomes, miR-224-5p, apotosis, autophagy

## Abstract

**Objective:** In this study, we focused on the potential mechanism of miRNAs carried by human umbilical cord mesenchymal stem cells-derived exosomes (hUCMSCs-exo) in breast cancer (BC).

**Methods:** RT-qPCR was conducted for the expression of miR-224-5p and HOXA5 in tissues and cells. After co-culture of exosomes and MCF-7 or MDA-MB-231 cells, the cell proliferation was observed by MTT and cell colony formation assay, while apoptosis was measured by flow cytometry. In addition, the expression of HOXA5 and autophagy pathway-related proteins LC3-II, Beclin-1 and P62 was detected by western blotting. And immunofluorescence was applied for detection of LC3 spots. The binding of miR-224-5p to HOXA5 was verified by the luciferase reporter gene assay and RNA-binding protein immunoprecipitation assay. Finally, *in vivo* experiment was performed to investigate the effect of miR-224-5p on BC growth.

**Results:** MiR-224-5p was up-regulated and HOXA5 was down-regulated in BC tissues and cells. HOXA5 was confirmed to be the target gene of miR-224-5p. MiR-224-5p carried by hUCMSCs-exo was able to promote the proliferation and autophagy of BC cells, while inhibited apoptosis. Bases on xenograft models in nude mice, it was also revealed that miR-224-5p carried by hUCMSCs-exo could regulate autophagy and contribute to the occurrence and development of BC *in vivo*.

**Conclusion:** MiR-224-5p carried by hUCMSCs-exo can regulate autophagy via inhibition of HOXA5, thus affecting the proliferation and apoptosis of BC cells.

## Introduction

Breast cancer (BC), as the most common malignant tumor, is the leading cause of malignant cancer-related death in women worldwide (Sun et al., 2017; Bray et al., 2018). Since the late 1970s, the global incidence of BC has been on the rise worldwide. Among malignant tumors in Chinese women, its incidence ranks first and its mortality is second only to lung cancer (Waks and Winer, 2019), which seriously affects health and life of women. However, the exact pathogenesis of BC remains unclear. In recent years, with the continuous improvement of diagnosis and treatment techniques of BC, its mortality still has no significant downward trend (Dihge et al., 2019). Nevertheless, understanding the histologic grades, lymph node involvement, and hormone receptor status, and detecting serum markers are all helpful for early diagnosis of BC, consequently improving prognosis, evaluating, and guiding for treatment (Radenkovic et al., 2014, 2019; Jurisic et al., 2015; Teufelsbauer et al., 2019). Malignant transformation of tumor cells involves multiple functional programming during which a number of gene products of therapeutic significance are induced, which can be used as molecular biomarkers and therapeutic targets to develop novel multi-target strategies to improve current cancer therapies and prevent disease recurrence (Mimeault and Batra, 2014). Therefore, a large number of scholars continuously search for molecular indicators for early diagnosis of BC, molecular targets for its treatment and molecular markers for judging its prognosis.

Surgical treatment, radiotherapy and chemotherapy are currently the main methods for treatment of BC. Stem cells recently have been found to exert a certain inhibitory effect on tumor cell growth (Leng et al., 2014). Mesenchymal stem cells (MSCs), derived from mesoderm, are pluripotent stem cells with the ability of self-renewal and multilineage differentiation, which are present in various tissues like bone marrow, blood, fat, placenta, umbilical cord, and skin (Almalki and Agrawal, 2016). Because of their effects on immunosuppression and tissue repair, MSCs are widely used in the treatment of a variety of diseases. Human umbilical cord mesenchymal stem cells (hUCMSCs), a kind of pluripotent stem cells present in neonatal umbilical cord tissue, which can differentiate into many kinds of cells. Based on their high cell content, strong proliferation ability and high differentiation potential, hUCMSCs have a broad prospect of clinical application. Exosomes refer to a kind of discoid vesicles with a diameter of 40–100 nm, containing RNAs and proteins. A variety of cells can secrete exosomes in both normal and pathological conditions. For example, MSCs can produce a large number of exosomes in resting or stress state. These MSC-derived exosomes have similar biological functions to MSCs, which can mediate intercellular communication (Zhu et al., 2012; Phinney et al., 2015).

MicroRNAs (miRNAs), a class of short non-coding RNAs (18–25 nucleotides), bind to the 3′ UTR of target mRNAs and thus act as post-transcriptional modulators of gene expression (Schickel et al., 2008) MiRNAs in BC have received significant attention. However, how to transport miRNAs into BC cells in a safe and efficient way is still an urgent problem to be solved. Exosomes are considered as novel nanomedicine carriers because of their perceived advantages like less cytotoxicity, strong targeting, low immunogenicity and large loading. At present, exosomes have successfully transported drugs such as small interfering RNA (siRNA), miRNA and paclitaxel to target cells (Santos et al., 2018; Takahasi et al., 2018). With the deepening of research, miRNAs, have been found to function in the occurrence and development of BC by affecting autophagy. For example, miR-30a can inhibit autophagy by negatively regulating the expression of Beclin1 (Zhu et al., 2009); miR-181a can inhibit autophagy in MCF-7 cells by binding with ATG5 (Tekirdag et al., 2013); miR-376b can inhibit starvation-related autophagy by down-regulating ATG4C (Korkmaz et al., 2012); and miR-221/222 leads to autophagic death of MCF-7 cells by PI3K-AKT-mTOR signaling pathway (Zhai et al., 2013).

MiR-224-5p is recently discovered to be associated with MSC-derived exosomes, tumor proliferation and metastasis. The expression of miR-224-5p is associated with original cisplatin resistance in ovarian papillary serous carcinoma (Zhao et al., 2014), and lncRNA FTH1P3 can mediate the growth and invasion of uveal melanoma cells through miR-224-5p (Zheng et al., 2017). Our previous study found that miR-224-5p inhibits autophagy by targeting Smad4 in MDA-MB-231 cells (Cheng et al., 2018). However, whether miR-224-5p can be transported by hUCMSCs-derived exosomes (hUCMSCs-exo) to affect autophagy in BC cells are not well clear. Here, we investigated the effect and mechanism of miR-224-5p carried by hUCMSCs-exo on BC cell proliferation and apoptosis, thus providing a new theoretical basis for the treatment of BC.

## Materials and Methods

### Collection and Processing of Clinical Samples

BC tissues and non-tumor adjacent normal tissues from 30 BC patients (mean age: 51.73 ± 9.66 years) admitted to our hospital from June 2016 to December 2017 were collected. The basic information of the patients was also recorded: age, gender, blood pressure, tumor stage, distant metastasis or not, and lymphatic metastasis or not. In all the patients, BC was the primary lesion and confirmed by pathological examination. Each patient has no history of major systemic disease and was not treated with chemotherapy or radiotherapy before surgery. Informed consent was obtained from each patient.

### Extraction and Identification of hUCMSCs-Derived Exosomes

HUCMSCs purchased from American Type Culture Collection (ATCC), and were cultured in FBS-free medium for 48 h, followed by collection of the supernatant. Centrifugation (2,000 *g*, 4°C for 30 min) was then performed to remove cell debris and apoptotic bodies and a 0.2 mm filter was used for filtration. Subsequently, the samples were first centrifugated at 110,000 *g*, 4°C for 70 min and then at 110,000 *g*, 4°C for 60 min. The collected exosomes were resuspended with phosphate buffer, and later were aliquoted and stored at −80°C.

In order to determine the characteristics of exosomes, the diameter and concentration of exosomes were analyzed by nanoparticle tracking analysis (NTA) using NanoSight NS300 (Malvern, UN) equipped with a 405 nm blue laser.

The morphology of exosomes was observed using a transmission electron microscope (TEM, Hillsboro Oregon, United States) (Tadokoro et al., 2013). Briefly, the exosomes were fixed with 4% paraformaldehyde and adsorbed to a carbon-coated grid. Then the copper grid was immersed in a 2% phosphotungstic acid solution for 30 s. After drying, the samples were examined under TEM at 80 keV. The expression of the marker proteins CD9, CD63, and HSP70 of the exosomes was detected by western blotting.

### Cell Transfection and Culture

Human BC cell lines MCF-7, MDA-MB-231, T47D, SK-BR-3, MDA-MB-435, and HCC1937, and human normal breast epithelial cells MCF10A were purchased from the Cell Culture Center of the Institute of Basic Medical Sciences, Chinese Academy of Medical Sciences. All cells were grown in media supplemented with 10% fetal bovine serum (Thermo Fisher Scientific) with culture conditions of 37°C and 5% CO_2_. MiR-224-5p mimic, NC mimic, miR-224-5p inhibitor, and NC inhibitor purchased from Guangzhou RiboBio Co., Ltd. (Guangzhou, China) were transfected into hUCMSCs, respectively, and the exosomes of each group were collected. MCF-7 or MDA-MB-231 cells were assigned into six groups: (1) Control: cells without any treatment; (2) Exo: cells were co-cultured with hUCMSCs-exo; (3) NC-exo: cells were co-cultured with exosomes transfected with NC mimics; (4) miR-224-5p-exo: cells were co-cultured with exosomes transfected with miR-224-5p mimics; (5) in-NC-exo: cells were co-cultured with exosomes transfected with NC inhibitor; (6) in-miR-224-5p-exo: cells were co-cultured with exosomes transfected with miR-224-5p inhibitor.

### MTT Assay

MCF-7 and MDA-MB-231 cells in logarithmic growth phase in each group were digested and counted. Then they were seeded in 96-well plates (2,000 cells/well) with five replicates for each sample. For adherent cells after treatment for 24, 48, and 72 h the media were aspirated from each group. Then the prepared MTT solution (Aladdin, United States) was added, followed by another 1-h incubation. The absorbance (490 nm) was measured by a microplate reader.

### Colony Formation Assay

The treated MCF-7 and MDA-MB-231 cells in each group were digested with trypsin to prepare single-cell suspension. The cell concentration was determined and adjusted to 1,000 cells/ml. Single-cell suspension was evenly seeded in sterile 6-well plates for forming colonies. The medium was changed every 3 days, and the colony formation of the cells was observed after 14 days.

### Flow Cytometry

The determination of apoptosis by flow cytometry was performed as shown in the previous report (Jurisic et al., 2011). Cells were centrifuged at 1,000 r/min for 5 min and then the supernatant was discarded. Subsequently, the cells were resuspended and counted. A total of 50,000–100,000 cells were taken and resuspended with 195 μl of Annexin V-FITC binding solution, followed by addition of 5 μl of Annexin V-FITC reagent. The cells were gently mixed with the reagent, and placed at room temperature for 10 min in the dark. After another 10 μl of propidium iodide staining solution was added and mixed gently, the samples were placed at room temperature for 10 min in the dark. Finally, the sample was detected by a flow cytometer.

### Immunofluorescence Staining

The treated MCF-7 and MDA-MB-231 cells in each group were seeded on coverslips. With cell confluency of about 70%, the coverslips were rinsed with PBS three times (10 min each time). Then the coverslips were fixed with 4% formaldehyde for 15 min and rinsed again with PBS. After that, 0.5% Triton X-100 was added for 10-min incubation, followed by rinsing step by PBS and 1-h blocking step using 100 mL/L calf serum albumin. The coverslips were then cultured with LC3 antibody (Abcam, Cambridge, United Kingdom; Dilution, 1:50) overnight at 4°C. After rinsing with PBS again, human FITC-labeled immunofluorescence antibody was added for 1-h incubation at 37°C. Finally, the coverslips were rinsed with PBS and distilled water, and, were mounted with glycerol. LC3 spots in each group were observed using a confocal microscope (Leica, Germany) with 400 × magnification.

### RNA-Binding Protein Immunoprecipitation Assay

Activated cells were collected and washed twice with PBS. Cells were cross-linked for 15 min by adding 10 mL PBS and formaldehyde with the final concentration of 0.01%. Then 1.4 mL of 2 mol/L glycine was added and mixed for 5 min, followed by centrifugation at 1,500 r/min for 5 min. The supernatant was discarded and then cells were washed twice with PBS again and lysed by RIPA. The cell lysates were equally assigned into two parts. Subsequently, one part with addition of 4 μg of AGO2 antibody (Abcam) was as the experimental group, while the other part with addition of the amount of normal rabbit IgG as the control group. Both groups were cultured overnight at 4°C, and then the cells were collected. RNA was extracted using TRIzol reagent and was reversely transcribed into cDNA. Finally, the expression of miR-224-5p and HOXA5 was detected with RT-qPCR

### Luciferase Reporter Gene Assay

The binding site of miR-224-5p to HOXA5 was predicted by software TargetScan. The 3’ UTR of the wild-type HOXA5 gene was cloned into a luciferase vector (HOXA5-WT), and the binding region of HOXA5 to miR-224-5p was mutated to obtain the mutant plasmid (HOXA5-MUT) (GenePharma, China). NC mimics or miR-224-5p mimics were co-transfected with HOXA5-WT/MUT into MCF-7 and MDA-MB-231 cells. Transfected pRL-TK was used as a standard. After 36 h of transfection, cells were harvested. The luciferase activity of MCF-7 and MDA-MB-231 cells was detected according to the instructions of luciferase activity assay kit. The experiment was repeated three times.

### RT-qPCR

Total RNA was isolated from cells and tissues using Trizol reagent (Invitrogen). A total of 500 ng of RNA was reversely transcribed into cDNA using the cDNA Transcription Kit (ABI). Transcription was subsequently carried out at 16°C for 30 min, followed by incubation at 42°C for 30 min and at 85°C for 5 min for enzyme inactivation. Rapid quantitative PCR was performed using SYBRH Select Master Mix (Invitrogen). The RT-qPCR reaction was performed using the following parameters: 95°C for 2 min, 40 cycles of 95°C for 15 s and 60°C for 30 s. With All results were standardized to the expression of GAPDH or U6 as an internal reference, the obtained experimental data was quantified using the 2^–Δ^ ^Δ^ ^*Ct*^ method. The primer sequences used were as follows: miR-224-5p, Forward, 5′-AGCATCCACGAGCAAGAGAC-3′ and reversely, 5′-GATGCTACTAGTGTGGCGGG-3′; U6, Forward, 5′-CTCGC TTCGGCAGCACA-3′ and reversely, 5′-AACGCTTCACGAAT TTGCGT-3′; HOXA5, Forward, 5′-GAAGCTGAGCAG TGAAGCCTAT-3′ and reversely, 5′-GACAACTGTGAGAGCC AGGTT-3′; GAPDH, Forward, 5′-AACGGATTTGGTCGTA TTG-3′ and reversely, 5′-GGAAGATGGTGATGGGATT-3′.

### Western Blot

Total protein was extracted with RIPA buffer (Sigma Aldrich, United States) from cells and tissues as previously reported (Son et al., 2019). The concentration of the total extracted protein was determined by a BCA protein assay kit (Bio-rad, United States). The proteins were boiled and separated using sodium dodecyl sulfatepolycylamide gel electrophoresis (SDS-PAGE). The separated proteins were then transferred to PVDF membranes (300 mA, 80 min), followed by blocking step using 10% TBS (Sigma Aldrich, United States). Subsequently the membranes were incubated with primary anti-rabbit monoclonal antibody CD9 (ab92726, Abcam), rabbit polyclonal antibody LC3-II (ab51520, Abcam), rabbit monoclonal antibody p62 (ab56416, Abcam), rabbit monoclonal antibody Beclin-1 (ab207612, Abcam), rabbit monoclonal antibody CD63 (ab8219, Abcam), rabbit monoclonal antibody HSP70 (ab181606, Abcam), and rabbit monoclonal antibody HOXA5 (ab140636, Abcam) at a dilution of 1:1,000 at 4°C overnight. Rabbit monoclonal antibody β-actin (ab8226, Abcam) is the internal reference protein. After incubation, the membranes were washed three times and then incubated with polyclonal goat anti-rabbit IgG (ab6721, Abcam; 1:2,000) secondary antibody conjugated to horseradish peroxidase (HRP) (Sigma Aldrich, United States) at room temperature for 1 h. Then the membranes were washed for several times. The proteins were developed by an enhanced chemiluminescence regent. Images were subsequently quantified with Image J (NIH).

### Establishment of Breast Cancer Xenografts in Nude Mice

Twenty nude mice were reared at constant temperature (25–27°C), constant humidity (25–50%), and specific pathogen-free (SPF). MCF-7 cells in logarithmic growth phase were prepared into single-cell suspension by trypsin digestion, followed by centrifugation at 1,000 r/min for 10 min. Then the precipitate was collected and washed twice with phosphate buffered saline (PBS) to prepare cell suspension with a density of 1 × 10^7^ cells/mL. A total of 200 μL of the suspension were aspirated with a sterile trocar and inoculated subcutaneously in the disinfected armpit skin of nude mice. After 10 days of inoculation, the mice were randomly divided into control group, Exo group, in-NC-exo group, and in-miR-224-5p-exo group, with five nude mice in each group. Reagents in each group were dissolved in 200 μL PBS and were injected every 2 days for a total of five injections. The long and short axes of the tumors were measured with a vernier caliper every 7 days to calculate tumor volume and draw tumor growth curve. All treatment was completed after 35 days, and all the mice were then sacrificed. Subsequently, the xenografts were removed and weighed. Each tumor tissue was equally assigned into 3 parts, with 2 parts stored in liquid nitrogen for subsequent detection, and the other part fixed in 10% neutral formalin and embedded in paraffin for subsequent immunohistochemical staining.

### Immunohistochemical Staining (IHC)

All tumor tissues were fixed in 10% neutral formalin and embedded in paraffin. IHC staining was performed according to the instructions of IHC kits with rabbit anti- Ki-67 (ab15580, Abcam) monoclonal antibodies. The tissues were developed with diaminobenzidine (DAB) (Tokyo Chemical Industry Co., Ltd., Japan) colorimetric solution, counterstained with hematoxylin, and mounted with neutral resin. Finally, the cell staining was observed under an optical microscope with 200× magnification.

### Statistical Analysis

SPSS 22.0 was applied for data analysis. The results were expressed as mean ± standard deviation (SD). Comparison between the two groups was analyzed by *t*-test. Multi-group comparison of data was carried out using the one-way ANOVA followed by least significant difference (LSD) *t*-test. Pearson analysis was used to analyze the correlation between the expression of miR-224-5p and HOXA5. Each experiment was repeated at least three times. *P* < 0.05 was considered statistically significant.

## Results

### Up-Regulation of miR-224-5p Expression and Down-Regulation of HOXA5 Expression in Breast Cancer

**FIGURE 1:**
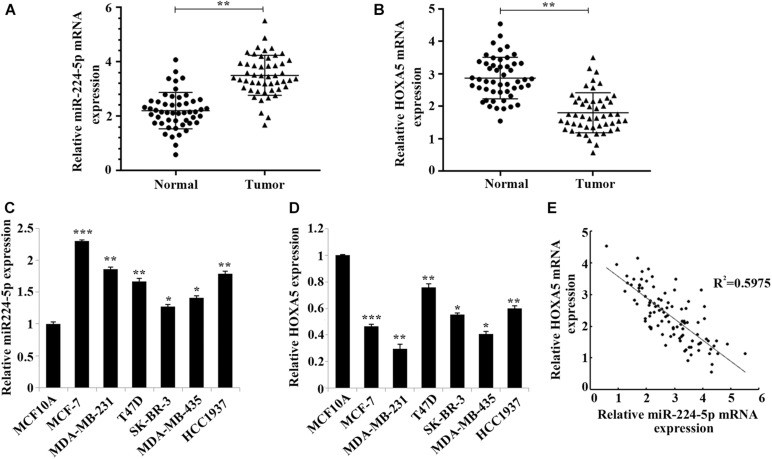
Negative correlation between miR-224-5p and HOXA5 expression in breast cancer. **(A,B)** RT-qPCR-based detection of miR-224-5p expression and HOXA5 expression in breast cancer tissues and non-tumor adjacent tissues; ^**^P < 0.01 vs. normal group. **(C,D)** RT-qPCR-based detection of miR-224-5p expression and HOXA5 expression in human breast epithelial cells MCF10A and human breast cancer cell lines MCF-7, MDA-MB-231, T47D, SK-BR-3, MDA-MB-435, and HCC1937; ^*^P < 0.05, ^**^P < 0.01 and ^***^P < 0.001 vs. MCF10A group. **(E)** Pearson correlation analysis between the expression of miR-224-5p and HOXA5.

The clinical characteristics of the 30 included BC patients are shown in Table 1. The results of RT-qPCR assay confirmed that the expression of miR-224-5p was significantly elevated while the expression of HOXA5 was significantly decreased in BC tissues (Figures 1A,B) and cells (Figures 1C,D). Pearson correlation analysis revealed a negative correlation between miR-224-5p expression and HOXA5 expression (Figure 1E). These results confirmed that miR-224-5p and HOXA5 may be involved in the occurrence of BC. Since the expression of miR-224-5p expression was higher while HOXA5 expression was lower in MCF-7 and MDA-MB-231 cells, these two cell lines were selected for the subsequent experiments.

**TABLE 1:** Breast cancer patients’ characteristic used in this study (*n* = 30).

**Characteristic**	***N* (%)**
Age (years)	
≥50	18 (60)
<50	12 (40)
TNMstage	
I	7 (23.3)
II	12 (40)
III	11 (36.7)
Lymph node metastasis	
No	12 (40)
Yes	18 (60)
Molecular classification	
Luminal A	3 (10)
Luminal B (HER-2 (-))	2 (6.7)
Luminal B (HER-2 (+))	25 (83.3)
Her-2 (+)	0
Basal-like	0

### Identification of hUCMSCs-Derived Exosomes

**FIGURE 2:**
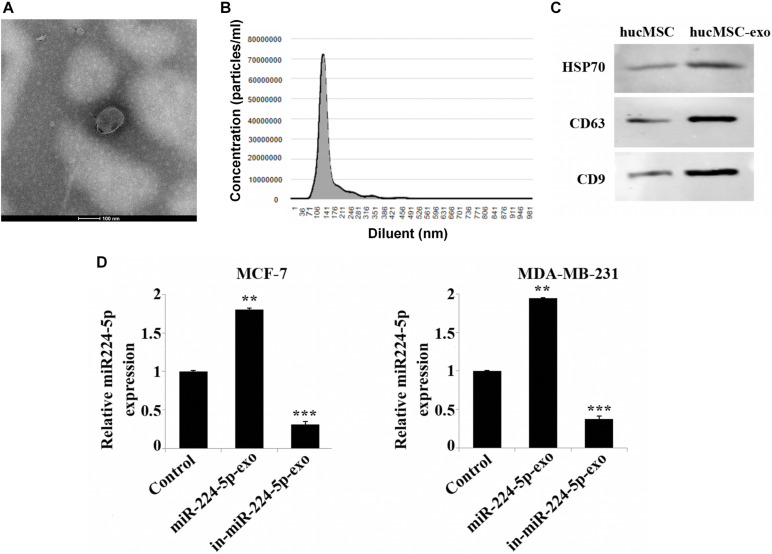
Extraction and identification of hUCMSCs-derived exosomes. **(A)** Observation of vesicle structure of hUCMSC-derived exosomes (hUCMSC-exo) by transmission electron microscopy; **(B)** Nanoparticle tracking analysis of the diameter range of hUCMSC-exo; **(C)** Western blotting-based detection of the expression levels of CD9, CD63 and HSP70 in hUCMSCs and hUCMSCs-exo; **(D)** RT-qPCR analysis of miR-224-5p expression. After transfection of miR-224-5p mimics and inhibitor into hUCMSCs, respectively, exosomes were collected and co-cultured with MCF-7 or MDA-MB-231 cells. Finally, the expression of miR-224-5p expression was detected by RT-qPCR. ^**^P < 0.01 and ^***^P < 0.001 vs. Control group.

To identify the functional effects of hUCMSCs-exo on MCF-7 and MDA-MB-231 cells, we first extracted and identified hUCMSCs-exo. Transmission electron microscopy confirmed the morphological characteristics of the exosomes as oval membranous vesicles (98 nm) (Figure 2A). And nanoparticle tracking analysis demonstrated that the diameter of most particles ranged from 49 to 160 nm (Figure 2B). In addition, the expression of protein markers CD9, CD63 and HSP70 of exosomes was up-regulated in the hUCMSC-exo compared with hUCMSCs (Figure 2C). Hsp70, CD9 and CD63 are the three protein markers of exosomes (Fernando et al., 2017). These results indicated a successful separation of exosomes. Further, after further overexpression or interference with the expression of miR-224-5p in hUCMSCs, exosomes were collected and then co-cultured with MCF-7 or MDA-MB-231 cells. As shown in RT-qPCR results, miR-224-5p expression was elevated in MCF-7 and MDA-MB-231 in the miR-224-5p-exo group, while was decreased in the in-miR-224-5p-exo group (Figure 2D). Collectively, it was proved that hUCMSCs-exo could transport miR-224-5p into BC cells to change miR-224-5p expression.

### Promotion of the Development of Breast Cancer Cells by miR-224-5p Carried by hUCMSCs-Derived Exosomes

**FIGURE 3:**
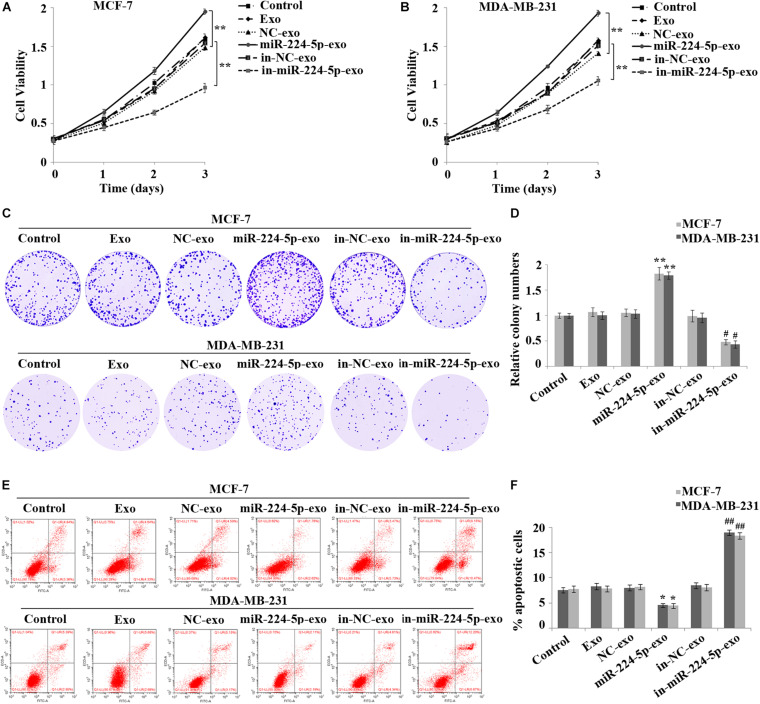
Promotion of proliferation and inhibition of apoptosis of breast cancer cells by miR-224-5p carried by hUCMSCs-derived exosomes. After transfection of miR-224-5p mimics and inhibitor and the negative controls into hUCMSCs, respectively, exosomes were collected and co-cultured with MCF-7 or MDA-MB-231 cells, followed by **(A,B)** MTT assay-based detection of cell proliferation; **(C,D)** Colony formation assay-based detection of cell viability; **(E,F)** Flow cytometry assay-based detection of cell apoptosis rate. ^**^P < 0.01 vs. NC-exo group; ^#^*P* < 0.05 and ^##^*P* < 0.01 vs. in-NC-exo group.

To determine whether miR-224-5p could be transported into BC cells via hUCMSCs-exo and thus affecting cell function, after transfection of miR-224-5p mimics and inhibitor into hUCMSCs, respectively, exosomes were collected and co-cultured with MCF-7 or MDA-MB-231 cells. As shown in the results of MTT (Figures 3A,B) and cell colony formation assay (Figures 3C,D), the proliferation rate and viability of cells in the miR-224-5p-exo group were significantly increased in MCF-7 or MDA-MB-231 cells compared with the NC-exo group, while the proliferation rate and viability in the in-miR-224-5p-exo group were significantly decreased compared with in-NC-exo. Flow cytometry (Figures 3E,F) showed a significant increase of apoptosis in the miR-224-5p-exo group, while a decrease in the in the in-miR-224-5p-exo group.

### Promotion of Autophagy in the Breast Cancer Cells by miR-224-5p Carried by hUCMSCs-Derived Exosomes

**FIGURE 4:**
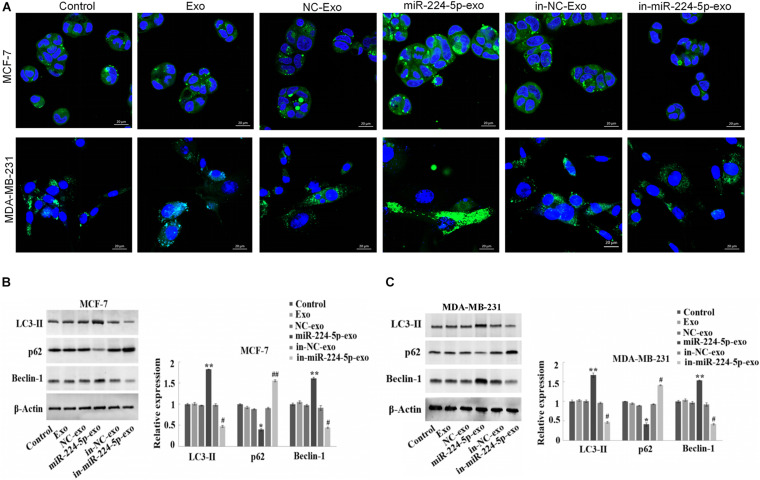
Promotion of autophagy in breast cancer cells by miR-224-5p carried by hUCMSCs-derived exosomes. After transfection of miR-224-5p mimics and inhibitor and the negative controls into hUCMSCs, respectively, exosomes were collected and co-cultured with MCF-7 or MDA-MB-231 cells, followed by **(A)** immunofluorescence staining-based determination of spot counting of LC3 (×400); **(B,C)** Western blotting-based detection of the expression of autophagy-related proteins LC3-II, Beclin-1 and P62. ^*^P < 0.05 and ^**^P < 0.01 vs. NC-exo group; ^#^*P* < 0.05 and ^##^*P* < 0.01 vs. in-NC-exo group.

The results of immunofluorescence staining indicated that in MCF-7 and MDA-MB-231 cells, the number of LC3 spots was significantly increased in the miR-224-5p-exo group compared with the NC-exo group, while was decreased in the in-miR-224-5p-exo group compared with the in-NC-exo group (Figure 4A). Western blot assay then showed a marked increase of the protein expression of LC3-II and Beclin-1 in MCF-7 and MDA-MB-231 cells and a reduction of p62 expression in the miR-224-5p-exo group compared with the NC-exo group, while opposite changes were found in the in-miR-224-5p-exo group (Figures 4B,C). These results confirmed that miR-224-5p could promote autophagy in BC cells.

### HOXA5 as a Target of miR-224-5p

**FIGURE 5:**
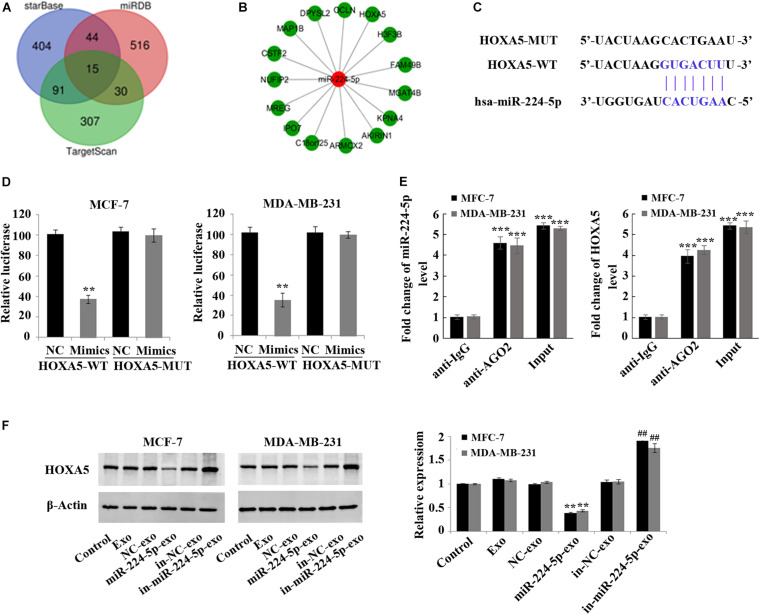
Negative regulation of HOXA5 expression in breast cancer cells by miR-224-5p carried by hUCMSCs-derived exosomes. **(A)** Prediction of target genes of miR-224-5p by StarBase, miRDB, and TargetScan; **(B)** fifteen potential target genes of miR-224-5p; **(C)** binding sites of miR-224-5p to HOXA5; **(D,E)** luciferase reporter gene assay and RNA binding protein immunoprecipitation assay to determine the binding of miR-219a-5p to HOXA5 in MCF-7 and MDA-MB-231 cells; **(F)** western blotting-based detection of the protein expression of HOXA5 in MCF-7 and MDA-MB-231 cells. ^**^P < 0.01 vs. NC-exo group; ^***^P < 0.001 vs. anti-IgG group; ^##^*P* < 0.01 vs. in-NC-exo group.

Fifteen downstream target genes (Figures 5A,B) regulated by miR-224-5p were predicted by starBase, miRDB and TargetScan. HOXA5, which is lowly expressed in BC, was also one of the target genes of miR-224-5p (Figure 5C). Luciferase reporter gene assay and RNA binding protein immunoprecipitation assay further proved that miR-224-5p targeted HOXA5 (Figures 5D,E). In addition, western blotting also confirmed that miR-224-5p could down-regulate the expression of HOXA5. In MCF-7 and MDA-MB-231 cells, the protein expression of HOXA5 was significantly decreased in the miR-224-5p-exo group compared with the NC-exo group, while was significantly increased in the in-miR-224-5p-exo group compared with the in-NC-exo group (Figure 5F).

### Promotion of Growth of Breast Cancer *in vivo* by miR-224-5p Carried by hUCMSCs-Derived Exosomes

**FIGURE 6:**
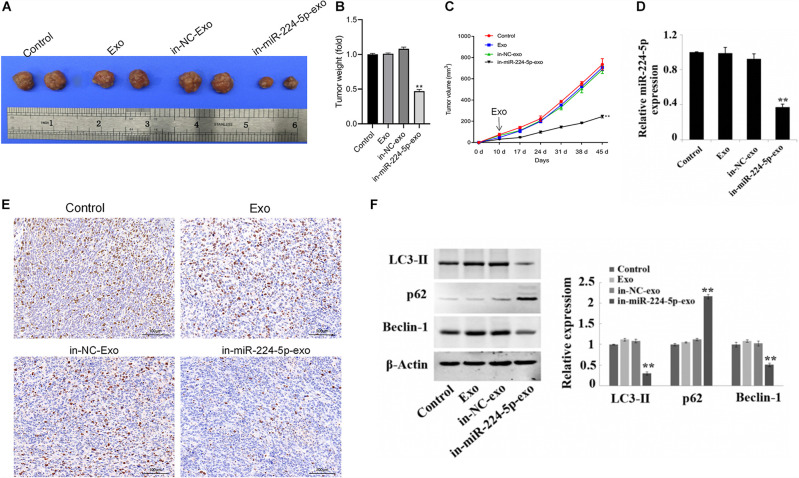
Regulation of proliferation and autophagy of breast cancer *in vivo* by miR-224-5p carried by hUCMSCs-derived exosomes. **(A)** Tumors of nude mice; **(B)** statistical graph of tumor weight; **(C)** statistical graph of tumor volume; **(D)** RT-qPCR analysis of miR-224-5p expression in tumor tissues; **(E)** immunohistochemistry-based determination of Ki67 expression in tumor tissues (×200); **(F)** western blotting-based detection of the protein expression of the autophagy-related proteins LC3-II, Beclin-1, and P62 in tumor tissues in nude mice. ^**^P < 0.01 vs. in-NC-exo group.

Xenograft models in nude mice were constructed by subcutaneous injection of MCF-7 cells in nude mice, followed by injection of saline (control group), Exo, in-NC-exo, and in-miR-224-5p-exo. The results showed that compared with the in-NC-exo group, the tumor tissue of mice in the in-miR-224-5p-exo group was significantly smaller (Figure 6A), and the tumor weight and volume of mice were decreased (Figures 6B,C). Injection of in-miR-224-5p-exo into the models decreased the expression of miR-224-5p and Ki67 (Figures 6D,E), decreased the protein expression of LC3-II and Beclin-1 and increased the protein expression of p62 in the tumor tissues (Figure 6F). These results confirmed that hUCMSCs-exo successfully transported low-expressed miR-224-5p into MCF-7 tumor-bearing mice to inhibit tumor growth and autophagy.

## Discussion

BC is one of the most common malignant tumors in women, posing a threat to physical and mental health of women (Anastasiadi et al., 2017). The traditional radiotherapy and chemotherapy have great damage to the normal cells of the body while killing tumor cells, so there is a great need to find a new treatment that can target tumor cells without harming normal tissues (Calle et al., 1978). MSCs have low immunogenicity, easy to culture and amplify *in vitro*, can migrate to tumor or inflammatory sites (Davatchi et al., 2016). MSCs are able to release a large number of cytokines to exert their tumor regulatory effects, such as interleukin-6 (IL-6), insulin-like growth factor 1 (IGF-1) or vascular epidermal growth factor (VEGF) (Wang et al., 2009; Jurisic, 2020). Therefore, MSCs have been widely used in treatment studies of many diseases. In recent years, it has been found that the regulatory effect of MSCs on tumors can also be mediated through exosomes secreted by MSCs. And MSCs have a powerful ability of exosomes secretion (Vakhshiteh et al., 2019). Exosomes are considered as a novel nano-scale drug carrier due to their low cytotoxicity, strong targeting, low immunogenicity and low-risk tumor formation (Hu et al., 2012; Semina et al., 2018). The study of miRNAs in tumor diseases is currently a hot spot, but how to transport miRNAs to BC cells in a safe and efficient way is still an urgent problem to be solved. Here, we used the exosomes secreted by hUCMSCs to transport miR-224-5p to promote the progression of BC, demonstrating that exosomes can be used as a safe and effective transport vector. In addition, the unique biological structure and function of exosomes provide a specific marker for the diagnosis of early BC, as well as a targeted strategy for its clinical treatment.

MiR-224-5p has been found to be associated with tumor proliferation and metastasis in recent years (Zhang et al., 2017). For example, miR-224 promotes drug resistance and recurrence of colorectal cancer by inducing epithelial-mesenchymal transition (Ling et al., 2016). But the functional mechanism of miR-224-5p in BC is not clear yet. In this study, we found that the expression level of miR-224-5p was significantly increased in human BC tissues and cell lines, suggesting the involvement of miR-224-5p in the occurrence and development of BC. Further, the binding site of miR-224-5p to 3′UTR of HOXA5 was predicted by the databases, suggesting that miR-224-5p may be involved in the development of BC by regulating HOXA5 expression. Previous studies have demonstrated that HOXA5 inhibits BC cell plasticity and stemness by reinforcing epithelial features (Teo et al., 2015). Here, we also found that HOXA5 was highly abundant in normal human breast epithelial cells and tissues, while its protein expression was low in human BC tissues and cell lines. In addition, both luciferase reporter gene assay and RNA binding protein immunoprecipitation assay confirmed that miR-224-5p could target and regulate HOXA5. Collectively, the above findings suggested that miR-224-5p could regulate the occurrence and development of BC by down-regulating the expression of HOXA5.

There has been a large body of related literatures concerning the association between miRNAs in exosomes and BC. For example, Naseri et al. (2018) have found that functionally active miRNA-142-3p inhibitors mediated by MSCs-derived exosomes reduce the tumorigenicity of BC *in vivo* and *in vitro*. Han et al. (2020) have confirmed that exosomes can reverse the resistance of trastuzumab in BC by delivering miR-567. To further verify the effect of miR-224-5p carried by exosomes on BC cell survival, MTT, colony assay and flow cytometry were performed. And the results demonstrated that hUCMSCs-exo could transport miR-224-5p into MCF-7 and MDA-MB-231 cells to promote the proliferation and survival of cancer cells and inhibit the decrease of their apoptotic rate. Ki-67 protein is a marker of tumor proliferation (Menon et al., 2019). Meanwhile, we found that the weight and volume of tumor were reduced and the expression of Ki67 was decreased after injection of hUCMSCs-exo with low miR-224-5p expression into BC tumor-bearing mice. These findings confirm that hUCMSCs-exo can transport miR-224-5p into BC cells to regulate BC development.

After determining the effect of miR-224-5p on the biological characteristics of BC cells, the specific mechanism of the effect became the emphasis of our subsequent investigation. Numerous reports have confirmed that autophagy plays a key role in various cellular events, including regulation of gene expression, growth and proliferation (Pietrocola et al., 2017; Soni et al., 2018). In recent years, with the continuous deepening of research, it has been found that miRNAs can affect autophagy during the occurrence and development of BC (Lv et al., 2017). For example, miR-30a, one of the first-discovered miRNAs involved in BC regulation, can inhibit autophagy by negatively regulating Beclin-1 expression in BC (Zhu et al., 2009). MiR-101 can effectively inhibit basal autophagy and rapamycin-mediated autophagy in MCF-7 cells (Frankel et al., 2011). MiR-181a can inhibit autophagy in MCF-7 cells by binding to ATG5 (Tekirdag et al., 2013). In addition, studies have also confirmed that miR-221/222 led to autophagic death of MCF-7 cells by participating in the PI3K/AKT/mTOR signaling pathway to inhibit P27Kip1 (Miller et al., 2008; Zhai et al., 2013). LC3-II, p62 and Beclin-1 are autophagy markers (Gómez-Sánchez et al., 2016; Runwal et al., 2019; Xu and Qin, 2019). In this study, after promoting the expression of miR-224-5p, significant increases of the number of LC3 autophagic spots and LC3-II and Beclin-1 expression and a decrease of p62 expression were found; inhibition of miR-224-5p achieved the opposite results. At the same time, similar findings were obtained in the xenograft models. Therefore, we speculate that miR-224-5p transported by the hUCMSCs-exo can promote autophagy by down-regulating the expression of HOXA5, thus ultimately promoting the development of BC. However, in previous studies, low expression of miR-224-5p has been found to promote BC cell autophagy and thus promote cell apoptosis (Cheng et al., 2018). This may be due to the effect of exosomes on the autophagy of BC cells (Xu et al., 2018), or due to the inconsistent results of the autophagy protein expression detected at different time points in the two studies. These assumptions require further experiments to confirmed.

## Conclusion

In summary, miR-224-5p is highly expressed in BC cells and tissues. In-depth mechanistic investigation confirms that miR-224-5p carried by hUCMSCs-exo regulates the autophagy of BC cell by down-regulating HOXA5, thus ultimately promoting the occurrence and development of BC. These results suggest the clinical application prospect of miR-224-5p carried by hUCMSCs-exo derived in the treatment of BC patients.

## Data Availability Statement

The original contributions presented in the study are included in the article/supplementary material, further inquiries can be directed to the corresponding author/s.

## Ethics Statement

The studies involving human participants were reviewed and approved by the Ethics Committee of Taizhou Central Hospital (Taizhou University Hospital) (2019-008). Written informed consent for participation was not required for this study in accordance with the national legislation and the institutional requirements.

## Author Contributions

YW, PW, LeZ, LiZ, and ZYL: study concept and design, drafting of the manuscript, and critical revision of the manuscript for important intellectual content. XC and ZL: analysis and interpretation of data. YW, PW, LeZ, XC, ZYL, LiZ, and ZL: acquisition of data, statistical analysis, administrative, technical, and material support, and study supervision. All authors have read and approved the manuscript.

## Conflict of Interest

The authors declare that the research was conducted in the absence of any commercial or financial relationships that could be construed as a potential conflict of interest.
